# Body electrical loss analysis (BELA) in the assessment of visceral fat: a demonstration

**DOI:** 10.1186/1475-925X-10-98

**Published:** 2011-11-10

**Authors:** Kim H Blomqvist, Jesper Lundbom, Nina Lundbom, Raimo E Sepponen

**Affiliations:** 1 Department of Electronics, Aalto University, PO Box 13340, 00076 Aalto, Finland; 2 Department of Radiology, University of Helsinki and HUS Radiology (Medical Imaging Center), PO Box 340, 00027 HUS, Finland

## Abstract

**Background:**

Body electrical loss analysis (BELA) is a new non-invasive way to assess visceral fat depot size through the use of electromagnetism. BELA has worked well in phantom measurements, but the technology is not yet fully validated.

**Methods:**

Ten volunteers (5 men and 5 women, age: 22-60 y, BMI: 21-30 kg/m^2^, waist circumference: 73-108 cm) were measured with the BELA instrument and with cross-sectional magnetic resonance imaging (MRI) at the navel level, navel +5 cm and navel -5 cm. The BELA signal was compared with visceral and subcutaneous fat areas calculated from the MR images.

**Results:**

The BELA signal did not correlate with subcutaneous fat area at any level, but correlated significantly with visceral fat area at the navel level and navel +5 cm. The correlation was best at level of navel +5 cm (R^2 ^= 0.74, P < 0.005, SEE = 29.7 cm^2^, LOOCV = 40.1 cm^2^), where SEE is the standard error of the estimate and LOOCV is the root mean squared error of leave-one-out style cross-validation. The average estimate of repeatability of the BELA signal observed through the study was ±9.6 %. One of the volunteers had an exceptionally large amount of visceral fat, which was underestimated by BELA.

**Conclusions:**

The correlation of the BELA signal with the visceral but not with the subcutaneous fat area as measured by MRI is promising. The lack of correlation with the subcutaneous fat suggests that subcutaneous fat has a minor influence to the BELA signal. Further research will show if it is possible to develop a reliable low-cost method for the assessment of visceral fat either using BELA only or combining it, for example, with bioelectrical impedance measurement. The combination of these measurements may help assessing visceral fat in a large scale of body composition. Before large-scale clinical testing and ROC analysis, the initial BELA instrumentation requires improvements. The accuracy of the present equipment is not sufficient for such new technology.

## Background

Accumulation of visceral fat (VF) has been shown to be important in the development of Type 2 diabetes and cardiovascular disease, while subcutaneous fat (SF) carries minor risks [1,2]. Excessive visceral fat may predispose even young children to these adult age diseases [3]. Therefore, VF and SF depots must be differentiated in interventional studies. The size of the visceral adipose depot can be accurately measured by magnetic resonance imaging (MRI) [4] or computed tomography (CT) [5], but the high measurement costs and CT's radiation dose make these methods unsuitable for large studies or repeated individual use. Anthropometric methods, such as waist circumference (WC) and waist-to-hip ratio (WHR), are frequently used as alternative indices for predicting VF related risks. However, these methods are known to be non-specific [6] because they include SF. Conventional bioelectrical impedance analysis (BIA) has also difficulties in measuring VF due to the influence of SF [7], and therefore various abdominal BIA methods have been introduced to measure VF more accurately [7-12]. Efforts have even been put into the use of ultrasound in the assessment of VF [13-17].

We are constructing an instrumentation to measure visceral adiposity with a radiofrequency coil, relying on the measurement of electrical losses in the subject through the use of electromagnetism. The idea of the measurement, called body electrical loss analysis (BELA), has been tested with phantom measurements [18]. The experiment does not expose the patient to ionizing radiation, and the strength of the magnetic field is below ICNIRP's (International Commission on Non-Ionizing Radiation Protection) guidelines [19] for general public exposure. Because of its simplicity, the BELA measurement could also be used to measure VF in children, which would be an important aid in preventive care [20].

The aim of this pilot study was to evaluate, how the BELA signal and the initial instrumentation perform in determining the size of the VF depot in humans, and to validate the results using MRI.

## Subjects and methods

**Figure 1:**
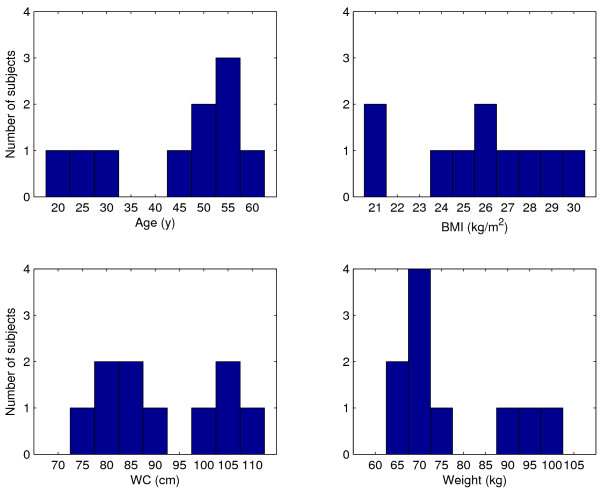
**Description of subjects**. The age, BMI, WC and weight histograms of the subjects.

The coordinating ethics committee of Helsinki University Central Hospital approved the study and an informed written consent was taken from the volunteers. All volunteers were measured with the BELA instrument and imaged with MRI within one week. Ten healthy volunteers (5 men, 5 women) covering the range of BMI of 21-30 kg/m^2 ^and the range of waist circumference of 73 to 108 cm were recruited from laboratory personnel and acquaintances. More descriptive data is shown in figure 1.

### Measurement of fat distribution by MRI

Fat distribution was measured on a clinical 1.5 Tesla MR imager (Avanto, Siemens, Erlangen, Germany) using a T1-weighted gradient echo sequence with selective fat excitation for optimal fat contrast [4]. A stack of sixteen axial image slices with 1 cm nominal thickness was centered at the L4/L5 intervertebral disk to determine the fat distribution at the waist level. The MR images were analyzed using a segmentation algorithm (SliceOmatic v4.3) to yield the areas of visceral and subcutaneous fat [4].

### BELA measurement

**Figure 2:**
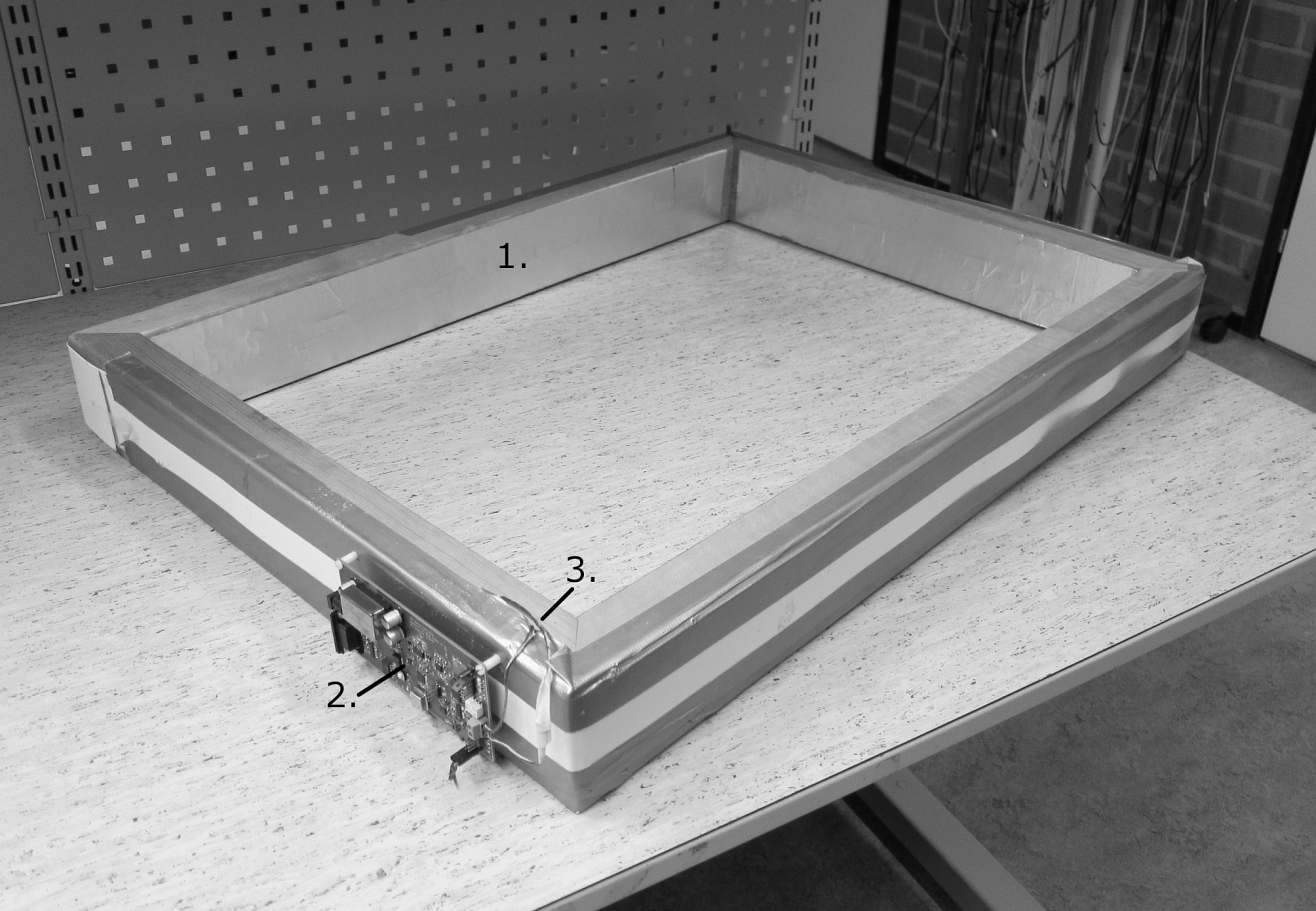
**BELA feasibility model**. A coil large enough for a human body to be surrounded at the abdomen. (1) An electrostatic shield, (2) measurement electronics, (3) multistrand litz wire [28].

During the BELA measurement, the volunteer crouches into the radio frequency coil - so that the coil surrounds the individual at the abdomen - and stands still hands over head, with shoes and belt removed. The apparatus is shown in figure 2. A time-varying magnetic field produced by the coil will induce so called eddy currents into the patient. These induced currents produce electrical losses in the patient, which can be observed as changes in the electrical resonance quality factor of the coil. Because the electrical conductivity of lean tissue is over ten times higher than that of fat tissue across the frequency range from 1 kHz to 1 MHz [21], lean tissue (e.g. muscle) will produce a larger change in the coil's quality factor.

By inducing variations in the excitation frequency, the major signal contribution can be altered. At higher frequencies, the signal tends to concentrate on the outer shell volume of the subject, allowing the assessment of fat distribution. For example, in an individual who has a small amount of VF, the loss changing rate as a function of frequency will also be small. This conclusion is based on the rough body models [18], where the trunk consists of three separate areas: interior (fatty or non-fatty), muscles and SF. From an electrical point of view, only the interior and the conductive volume (of muscles) surrounding the interior interact in the BELA measurement. The influence of SF is considered to be small because of its low conductivity.

The loss changing rate is calculated with the following equation

(1) $$m_{\text{loss}}=\frac{\Delta V_{\text{i,H}}-\Delta V_{\text{i,L}}}{f_{\text{H}}-f_{\text{L}}}$$

where Δ*V*_i,H _and Δ*V*_i,L _are the measured voltage changes across the coil between empty and loaded coil at high and low (*f*_H _and *f*_L_) frequencies, respectively. The subscript *i *indicates the in-phase signals [18].

In this study the BELA measurements were taken at 103 kHz and 185 kHz frequencies at navel level, navel +5 cm and navel -5 cm. All measurements were repeated five times at every level. In addition, this session was repeated for one subject on three consecutive days.

The effect of the positioning was studied by taking measurements when standing three centimetres forward, rightward and leftward from the marked standing position (the middle of the coil). This test was repeated ten times for every positioning.

### Statistical analysis

Data were analyzed using the Pearson product-moment correlation coefficient and leave-one-out style cross-validation. The repeatability of the BELA signal was estimated as

(2) $$\Delta m_{\text{loss}}=\frac{m_{\text{loss,max}}-m_{\text{loss,min}}}{2}=\frac{\sigma_{\text{H}}+\sigma_{\text{L}}}{f_{\text{H}}-f_{\text{L}}}$$

where *σ*_H _and *σ*_L _are the standard deviations of the measured voltage changes at *f*_H _and *f*_L_, respectively. The effect of the frequency measurement was insignificant (< 5 · 10^-3^) and is thus ignored in equation 2.

## Results

**Figure 3:**
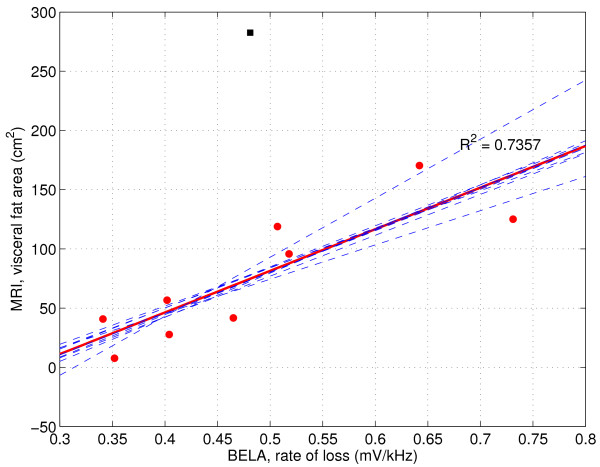
**BELA signal compared with VF area in MRI**. Linear regression (bold line) between the loss changing rate measured as voltage per frequency and VF area measured in the MR image at navel +5 cm level in nine volunteers. Dashed lines are multiple regression lines of leave-one-out style cross validation. One volunteer, marked with ■, had an exceptionally large amount of visceral fat, which the BELA could not recognize.

**Figure 4:**
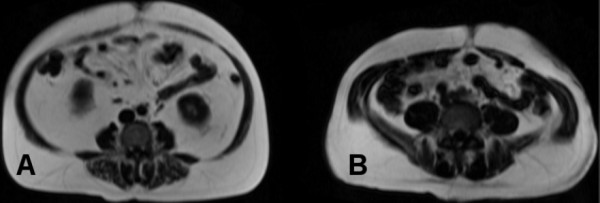
**Axial MR images from two volunteers**. One volunteer had an exceptionally large amount of VF (A) but smaller amount of SF than a volunteer with a reasonable amount of VF (B). The relative amount of conductive tissue (muscle) compared with poorly conductive fatty tissue is so small in (A) that the BELA could not differentiate VF from VF+SF.

The calculated correlation coefficients between BELA and MRI at three different levels of navel are shown in tables 1 and 2. The measured mean values of the loss changing rates (*m*_loss_) did not correlate with SF in MRI at any level (R^2 ^< 0.14). Significant correlations between *m*_loss _and MRI were found for VF (R^2 ^= 0.74, P < 0.005) at the navel +5 cm level and (R^2 ^= 0.43, P < 0.05) at the navel level. Figure 3 presents the scatter plot of the best correlation (SEE = 29.7 cm^2^, LOOCV = 40.1 cm^2^) between the *m*_loss _and VF area. SEE is the standard error of the estimate and LOOCV is the root mean squared error of leave-one-out style cross-validation. One measurement point, marked with a square, was exceptional and thus ignored in the calculations of correlation coefficients, see below figure 4A.

**Table 1:** Correlation (R^2^) of the loss changing rate (*m*_loss_) to the VF, SF, VF/SF and SF/VF at different levels

Level	VF	SF	VF/SF	SF/VF
Navel +5 cm	0.74, P < 0.005	0.005	0.38, P < 0.05	0.35, P < 0.05
Navel	0.43, P < 0.05	0.14	0.06	0.03
Navel -5 cm	0.19	0.002	0.08	0.19

**Table 2:** Correlation (R^2^) of the loss changing rate (*m*_loss_) to the BMI and WC at different levels

Level	BMI	WC
Navel +5 cm	0.33, P < 0.05	0.75, P < 0.005
Navel	0.35, P < 0.05	0.74, P < 0.005
Navel -5 cm	0.32	0.68, P < 0.005

The average estimate of the repeatability of the measured loss changing rates, observed through the study, was ±9.6 %. The effect of the standing position (center or off-center) was ±2.5 % at *f*_L _and ±1.5 % at *f*_H_. The repeatability was also studied in three different sessions in consecutive days for one subject. The results were 0.60 ± 0.03, 0.61 ± 0.01 and 0.57 ± 0.01 mV/kHz (mean ± SD) at navel level, navel +5 cm and navel -5 cm, respectively.

## Discussion

The BELA signal - the loss changing rate as a function of frequency (*m*_loss_) - did not correlate significantly with the amount of subcutaneous fat and, as had been predicted from the phantom study [18], tended to correlate with the amount of visceral fat. Comparison of the correlations found in this study to those found for a much simpler method waist circumference (WC) [22,23] do not suggest much advantage for the BELA measurement over WC. However, both the sensitivity and specificity of WC tend to weaken in individuals over 40 years of age [23], likely because of the accumulation of SF. The low sensitivity of BELA for SF could make it more specific in this age group.

The comparison of the BELA correlations to those found for abdominal BIA and ultrasonography (US) do not show much advantage either. However, large scale studies in abdominal BIA [9,10,12] and US [13] do not report receiver operating characteristics (ROC) graphs, which we think are important when the performance of the method is estimated. Furthermore, one may question the performance of the bioelectrical impedance measurement in the abdominal BIA [24], because the final analysis commonly includes several additional parameters like sex, age [11] and body shape [8,10,11], or WC [12]. The nature of the eddy currents may justify including WC in the BELA analysis, but there is no need to use the other parameters often combined with BIA. Thus, in some groups of individuals, BELA will potentially perform better than BIA. BELA would also be more convenient in medical check-ups and repeated individual use than US, because BELA measurements do not need medical personnel to perform the measurement or interpret the results.

The loss changing rate *m*_loss _is a relative quantity. It does not include information on the bulk losses caused by the subject but rather the rate of loss as a function of frequency. Based on this, we assumed that even if larger subjects introduce more losses than smaller ones, *m*_loss _would not be sensitive to the body size. However, a strong correlation to WC at all levels suggests that *m*_loss _is sensitive to body geometrics, which has to be targeted in further studies to make sure that the sensitivity to changes in fat content is sufficiently larger than the sensitivity to changes in WC.

The BELA setup used in this study was similar to that used in [18], except the doubled field strength (1V across the coil) and the electrostatic shield made to cover the coil's inner surface. By doubling the field strength, the signal from human subjects corresponded to what was observed with phantoms in [18]. This change was necessary, because at low frequency, the losses would have been too small. The electrostatic shield was included because, in human subjects but not in phantoms, losses were observed to be somewhat higher without shielding. The shield's purpose is to reduce capacitive coupling with the subject inside the coil. Therefore, only the inner surface of the coil was shielded. Shielding the outer surface would only reduce the coil's quality factor unnecessarily.

The losses were measured at 103 kHz and 185 kHz frequencies. The frequency selection is not critical and therefore these frequencies were not tuned precisely to some more intuitive frequencies like 100 kHz and 200 kHz by using trimmer capacitors but discrete components. It is important to have two separate frequencies low enough so that the wave length is clearly above the subject's diameter. Moreover, frequencies from 100 kHz to 200 kHz are low enough to give signal also from the interior of the subject. This was tested with air core and filled phantoms in [18] - a similar test than in [25].

One of the volunteers had an exceptionally large amount of VF, which the BELA measurement could not differentiate. The axial MR image, shown in figure 4A, indicated that in this volunteer, the VF and SF pools came into close contact with each other due to very thin or near non-existent abdominal muscles, thus significantly reducing the conductive volume (of muscle) between SF and VF pools. Since the BELA measurement needs an electrically conductive volume between the SF and VF pools, the measurement could not differentiate between the two fat pools, leading to underestimation of the VF and delaying possible treatment.

Individuals who do not exercise and maintain normal BMI through diet only may have a considerable proportion of VF. This "slim but fat inside" or thin-on-the-outside fat-on-the-inside (TOFI) body constitution carries a metabolic risk [26] and needs to be recognized. For BELA to perform well in a large range of body compositions, additional parameters are needed to give more information on the total conductivity of the measured area. Combining bioelectrical impedance measured at the waist level with BELA may allow a more reliable assessment of VF across a large range of body compositions. This combination of measurements should give enough information on subject's fat distribution so that TOFIs and cases similar to the one shown in figure 4A can be correctly classified. Practically, in these subjects, bioelectrical impedance should show exceptionally large impedance while the rate of loss remains small.

Both the accuracy and resolution of the BELA instrumentation need to be improved before performing evaluations in larger and more heterogeneous test groups. The measured voltage changes across the coil map to changes in the coil resistance. This change of resistance is very small, below mΩ at low frequency [18], which makes the measurement challenging. To improve the accuracy, we have developed a new pre-amplifier especially with the BELA measurement in mind [27]. The usage of this amplifier allows reducing the signal processing and provides higher gains in an analog front-end design. In this study, the accuracy and repeatability was limited by 10-bit analog-to-digital converter, which had an absolute accuracy of ±2 LSB. With 1.1V voltage reference, this is ±2.15 mV which corresponds the average standard deviations of the measured voltage changes through the study, 1.71 mV and 1.89 mV for low and high frequencies, respectively. Ten times better accuracy should be achievable which would suffice for clinical measurements. In addition, the average relative difference between the *m*_loss _at different levels of navel was only ~ 3.3 %, and the effective slice thickness of the initial version of the BELA setup was ~ 5 cm. To achieve a thinner slice, and thus better spatial resolution, the form of the magnetic field has to be changed. Work is now under progress towards a new instrumentation that will allow more extensive clinical testing.

## Conclusions

This paper demonstrates the potential of the body electrical loss analysis (BELA) in the assessment of visceral fat (VF) accumulation. The novel quantity *m*_loss _measured by BELA correlated significantly with the VF but not with the subcutaneous fat (SF) area as measured by MRI at the navel level and navel +5 cm. The lack of correlation with SF suggests that SF has a minor influence to the BELA signal allowing more VF specific measurement. Further research will show if it is possible to develop a reliable low-cost method for the assessment of VF either using BELA only or combining it, for example, with bioelectrical impedance measurement.

## Competing interests

The authors declare that they have no competing interests.

## Authors' contributions

KB constructed the BELA measurement setup, made the measurements, performed the statistical analysis and drafted the manuscript. JL carried out the MRI measurements, performed the segmentation and helped in the statistical analysis. NL designed and coordinated the MRI measurements, and helped to draft the manuscript. The original idea of the BELA measurement comes from RS who participated in the design of the study and revised the manuscript critically. All authors read and approved the final manuscript.
